# Supplementary material for: Serum coating enables feeder-free culture of naive human pluripotent stem cells preserving developmental potential
Source: EMBO J. 2026 Mar 12;45(8):2831–67. doi: 10.1038/s44318-026-00714-2 (PMC13083898; doi:10.1038/s44318-026-00714-2)
Supplement: Supplementary file 1 — Table EV1 [file 44318_2026_714_MOESM1_ESM.docx]

**Table EV1: Primers used in this study**

| **Group** | **Target** | **Sequence** |
| --- | --- | --- |
| Martello | POU5F1_FW  POU5F1_RW | GTGGAGGAAGCTGACAACAA  ATTCTCCAGGTTGCCTCTCA |
|  | NANOG_FW  NANOG_RV | CAAAGGCAAACAACCCACTT  TCTGCTGGAGGCTGAGGTAT |
|  | TFCP2L1_FW  TFCP2L1_RW | GGAGTTCCAGCCATGCTCTT  CCTGCTTGAAGATGGGCAGA |
|  | KLF4 _FW  KLF4 _RV | CCCAATTACCCATCCTTCCT  CAGGTGTGCCTTGAGATGG |
|  | KLF17_FW  KLF17_RV | CACACAGGTGAGAGGCCATA  ACTCGGTGTGTACCCGCATA |
|  | OTX2_FW  OTX2_RV | GAGAGGAGGTGGCACTGAAA  TGACCTCCATTCTGCTGTTG |
|  | ZIC2_FW  ZIC2_RV | CATGCACGTCCACACCTC  CTCATGGACCTTCATGTGCTT |
|  | SALL2_FW  SALL2_RV | GCACGAATCCGAGAGGAG  TCACCATTACAGGAGGGTCAG |
|  | CDH2_FW  CDH2_RV | CGTGGTCAAACCAATCGAC  AACAGACACGGTTGCAGTTG |
|  | GATA3_FW  GATA3_RV | ACAGAAGGCAGGGAGTGTGT  TCCGTTCATTTTGTGATAGAGC |
|  | GATA2_FW  GATA2_RV | GACTACAGCAGCGGACTCTT  GCCTTCTGAACAGGAACGAG |
|  | IGFBP3_FW  IGFBP3_RV | CCTGCCGTAGAGAAATGGAA  AAGGGCGACACTGCTTTTT |
|  | BAMBI_FW  BAMBI_RV | GAGGCCTCAGGACAAGGAAA  CCCGGAACCACAACTCTTT |
|  | SOX1_FW  SOX1_RV | GCTGACACCAGACTTGGGTTT  CCCCTCGAGCAAAGAAAACG |
|  | T_FW  T_RV | GCCAGATCATGCTGAACTCC  AAGCTTTTGCAAATGGATTGT |
|  | SOX17_FW  SOX17_RV | ACGCCGAGTTGAGCAAGA  TCTGCCTCCTCCACGAAG |
|  | GAPDH_FW  GAPDH_RV | GCCTCAAGATCATCAGCAATGC  CGCCACAGTTTCCCGGAG |
| Leeb | KLF4_FW  KLF4_RV | CCCACATGAAGCGACTTCCC  CAGGTCCAGGAGATCGTTGAA |
|  | ZIC2_FW  ZIC2_RV | CATGCACGGTCCACACCTC  CTCATGGACCTTCATGTGCTT |
|  | ACTB_FW  ACTB_RV | CCAACCGCCGAGAAGATGA  CCAGCGGCGTACAGGGATAG |
