# Supplementary material for: Serum coating enables feeder-free culture of naive human pluripotent stem cells preserving developmental potential
Source: EMBO J. 2026 Mar 12;45(8):2831–67. doi: 10.1038/s44318-026-00714-2 (PMC13083898; doi:10.1038/s44318-026-00714-2)
Supplement: Supplementary file 2 — Table EV2 [file 44318_2026_714_MOESM2_ESM.docx]

**Table EV2: Primary antibodies used in this study**

| **Group** | **Target** | **Conjugate** | **Supplier** | **Catalog ID** | **IF dil.** |
| --- | --- | --- | --- | --- | --- |
| Martello | KLF17 | - | Atlas Antibodies | HPA024629 | 1:250 |
|  | OCT4 (C-10) | - | Santa Cruz | sc-5279 | 1:300 |
|  | NANOG | - | Cell Signaling | D73G4 | 1:100 |
|  | TFCP2L1 | - | R&D Systems | AF5726 | 1:250 |
|  | GATA3 | - | R&D Systems | AF2605 | 1:100 |
| Leeb | KLF17 | - | Atlas Antibodies | HPA024629 | 1:333 |
|  | OCT4 (C-10) | - | Santa Cruz | sc-5279 | 1:50 |
|  | SUSD2 (W5C5) | APC | BioLegend | 327408 | 1:100 |
|  | KLF5 (G-7) | - | Santa Cruz | sc-398470 | 1:50 |
|  | H3K27me3 | - | Sigma-Aldrich | 07-449 | 1:500 |
|  | GATA3 | - | Invitrogen | MA1028 | 1:50-100 |
|  | GATA4 | - | Invitrogen | 14-9980-82 | 1:200 |
|  | TROP2 | AF488 | Bio-Techne | FAB650G | 1:100 |
| Pasque | KLF17 | - | Abcam | HPA024629 | 1:300 |
|  | OCT4 (C-10) | - | Santa Cruz | sc-5279 | 1:100 |
|  | NANOG | - | BD Bioscience | 560482 | 1:50 |
|  | GATA3 |  | R&D System | AF2605 | 1:100 |
|  | SOX17 | - | R&D System | AF1924 | 1:100 |
|  | SUSD2 | PE | Biolegend | 327406 | 1:50 |
|  | GATA2 | - | Sigma-Aldrich | WH0002624M1 | 1:100 |
|  | NANOG | - | Abcam | ab21624 | 1:100 |
| Zylicz | NANOG | - | Abcam | ab173368 | 1:250 |
|  | GATA3 | - | Abcam | ab199428 | 1:250 |
|  | GATA4 | - | eBioscience | 14-9980-80 | 1:250 |
| Rivron | OCT4 (C-10) | - | Santa Cruz | sc-5279 | 1:200 |
|  | CDX2 | - | Abcam | ab76541 | 1:1000 |
|  | GATA4 | - | Invitrogen | 14-9980-82 | 1:400 |
