# Supplementary material for: Serum coating enables feeder-free culture of naive human pluripotent stem cells preserving developmental potential
Source: EMBO J. 2026 Mar 12;45(8):2831–67. doi: 10.1038/s44318-026-00714-2 (PMC13083898; doi:10.1038/s44318-026-00714-2)
Supplement: Supplementary file 16 — Expanded View Figures [file 44318_2026_714_MOESM16_ESM.pdf]

Expanded View Figures

Figure EV1. Naive hPSCs on serum coating express key markers and can be genetically engineered.

(**A**) Top: Immunostaining for general pluripotency (OCT4 and NANOG) and naive (KLF17 and TFCP2L1) markers of naive HPD06 and HPD03 hiPSCs cultured on MEFs or serum coating at the 4th passage. Complete view of Fig. 1B. Scale bars: 100 µm. Representative images of two independent experiments are shown. Bottom: Mean fluorescence intensity quantification for general pluripotency (OCT4 and NANOG) and naive (KLF17 and TFCP2L1) markers of naive HPD06 and HPD03 hiPSCs cultured on MEFs or serum coating at the 4th passage. At least 700 nuclei from five randomly selected fields from two independent experiments were analysed for each cell line under different conditions. The box plot indicates the 25th, 50th and 75th percentiles. Two-sided unpaired Student's t-test of the means of independent experiments. (**B**) Immunostaining for general pluripotency (OCT4 and NANOG) and naive (KLF17 and SUSD2) markers of naive H9 and Shef6 hESCs, and SIG-1 hiPSCs stably cultured on MEFs or serum coating. Complete view of Fig. 1B. Scale bars: 100 µm. Representative images of two independent experiments are shown. (**C**) Gene expression analysis by RT-qPCR of general (*POU5F1* and *NANOG*), naive (*TFCP2L1*, *KLF4* and *KLF17*), and primed (*OTX2* and *ZIC2*) pluripotency markers in naive HPD06 and HPD03 hiPSCs on MEFs or serum coating at the 4th passage when plated at a low density. Bars indicate the mean ± SEM of technical replicates shown as dots from $n = 4$ independent experiments for primed hiPSCs. Technical replicates from $n = 2$ independent experiments for naive hPSCs are shown as dots. Two-sided unpaired Student's t-test. (**D**) Representative gating strategy to evaluate marker positivity in naive hPSCs by flow cytometry. Selected sub-populations are shown from left to right. First, the cell population was distinguished from cell debris (left panel). Singlets were chosen from the cell population, and live cells among singlets were selected, followed by the gating of marker-positive cells using the unstained negative control (right panel). This gating strategy corresponds to Figs. 1D and 5C, and EV4F. (**E**) Growth rate of naive HPD06 and HPD03 hiPSCs cultured on MEFs or serum coating when plated at a low density over the first 4 passages of the conversion. Bars indicate the mean ± SEM of technical replicates shown as dots from $n = 2$ independent experiments. Two-way repeated measures ANOVA. (**F**) Top: Representative AP staining images after clonal assay of naive HPD06 and HPD03 hiPSCs cultured on MEFs or serum coating when plated at a low density at the 4th passage. Bottom: Quantification of the relative number of AP-positive pluripotent colonies counted per well. Technical replicates from $n = 2$ independent experiments are shown as dots. Two-sided unpaired Student's t-test. (**G**) Morphologies (top) and fluorescence (bottom) of naive HPD06 hiPSCs transfected with an EGFP-piggyBAC 24 h post-transfection (left) and after selection and stable culture (bottom). Scale bars: 200 µm. Representative images of two independent experiments are shown. Source data are available online for this figure.

 © The Author(s)

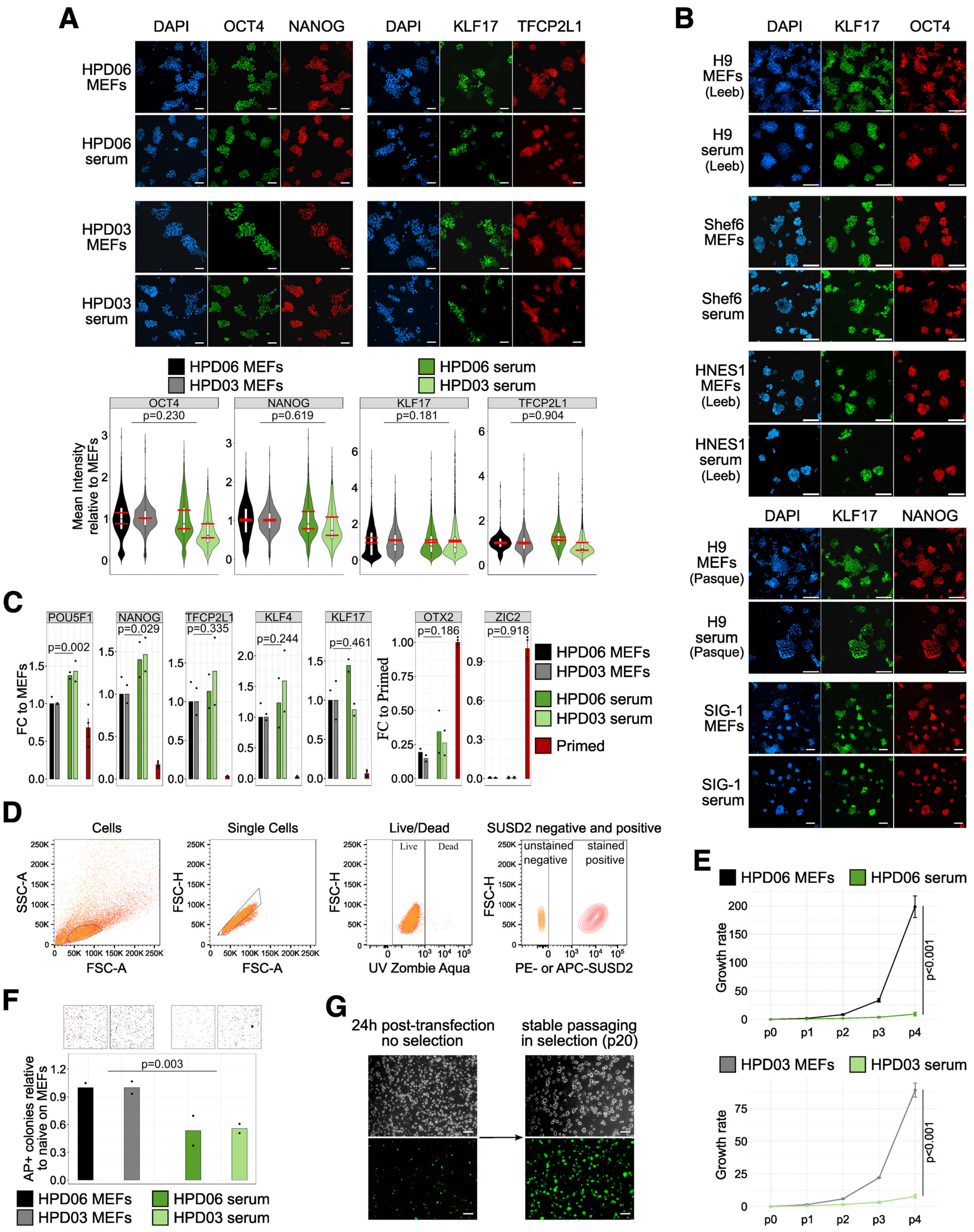
A
DAPI
OCT4
NANOG
DAPI
KLF17
TFCP2L1
HPD06 MEFs
HPD06 serum
HPD03 MEFs
HPD03 serum
HPD06 MEFs
HPD03 MEFs
HPD06 serum
HPD03 serum
OCT4 p=0.230
NANOG p=0.619
KLF17 p=0.181
TFCP2L1 p=0.904
Mean Intensity relative to MEFs
B
DAPI
KLF17
OCT4
H9 MEFs (Leeb)
H9 serum (Leeb)
Shef6 MEFs
Shef6 serum
HNES1 MEFs (Leeb)
HNES1 serum (Leeb)
DAPI
KLF17
NANOG
H9 MEFs (Pasque)
H9 serum (Pasque)
SIG-1 MEFs
SIG-1 serum
C
POU5F1 p=0.002
NANOG p=0.029
TFCP2L1 p=0.335
KLF4 p=0.244
KLF17 p=0.461
OTX2 p=0.186
ZIC2 p=0.918
FC to MEFs
FC to Primed
HPD06 MEFs
HPD03 MEFs
HPD06 serum
HPD03 serum
Primed
D
Cells
Single Cells
Live/Dead
SUSD2 negative and positive
SSC-A
FSC-A
FSC-H
Live
Dead
UV Zombie Aqua
unstained negative
stained positive
PE- or APC-SUSD2
E
HPD06 MEFs
HPD06 serum
Growth rate
p<0.001
HPD03 MEFs
HPD03 serum
p0
p1
p2
p3
p4
F
p=0.003
AP+ colonies relative to naive on MEFs
HPD06 MEFs
HPD03 MEFs
HPD06 serum
HPD03 serum
G
24h post-transfection no selection
stable passaging in selection (p20)

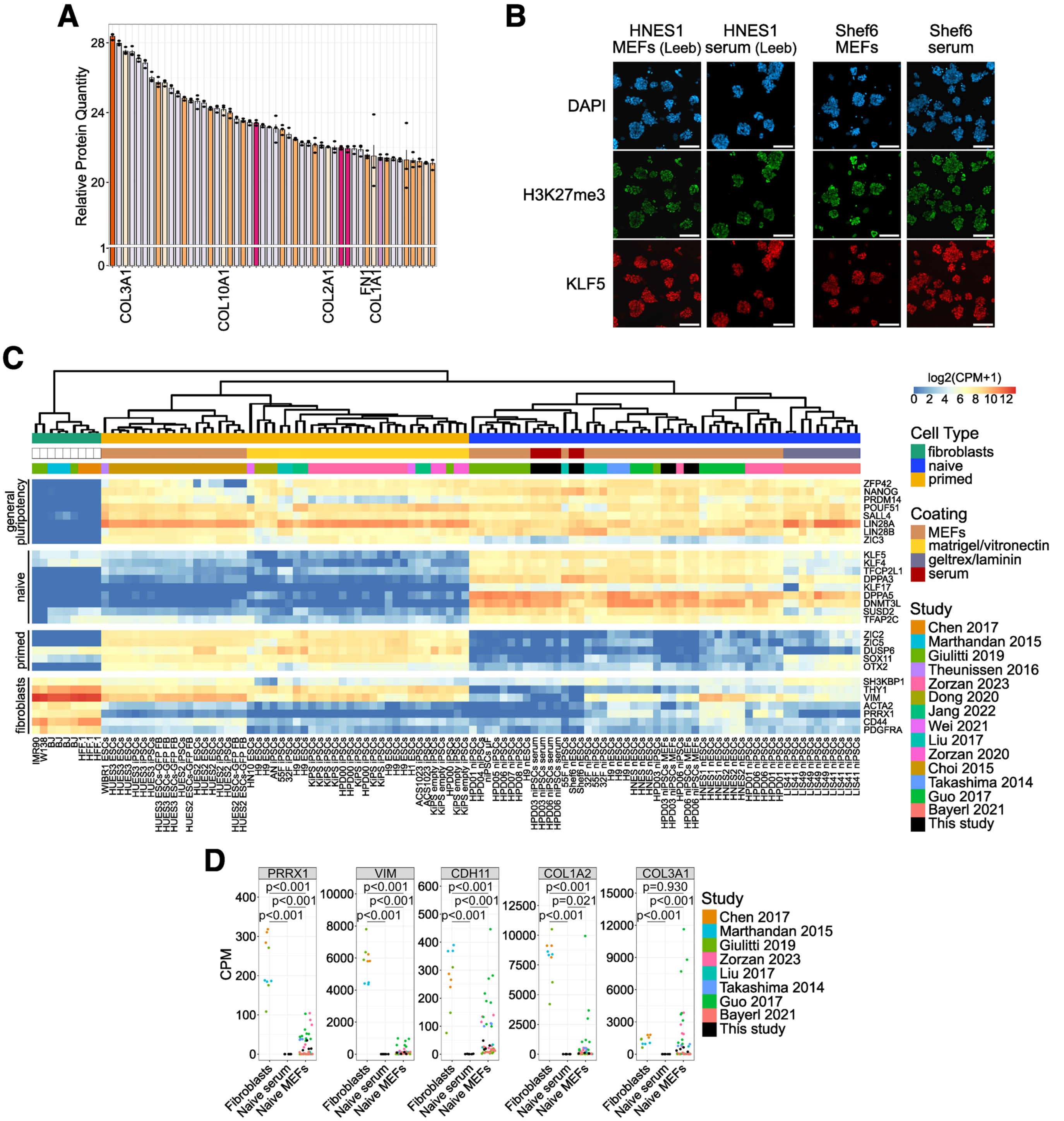

Figure EV2. Naive hPSCs on serum coating retain H3K27me3 marks and do not show contamination of MEFs transcripts.

(**A**) Top 50 most abundant proteins in one representative batch of serum coating. Detected Collagens and Vitronectin shared between 5 different serum coating batches are highlighted. Bars indicate the mean ± SEM of $n = 3$ technical replicates shown as dots. (**B**) Immunostaining for H3K27me3 and KLF5 of naive HNES1 and Shef6 hESCs stably cultured on MEFs or serum coating. Scale bars: 100 µm. Representative images of two experiments are shown. (**C**) Heatmap of general pluripotency, naive, primed, and fibroblasts genes in naive HPD06 and HPD03 hiPSCs lines and Shef6 hESCs stably cultured on MEFs or serum coating and in published fibroblasts, primed hPSCs and naive hPSCs. Extended version of Fig. 3D. (**D**) Absolute expression (CPM) of fibroblasts markers from Fig. 3D and others in naive HPD06 and HPD03 hiPSCs, and Shef6 hESCs stably cultured on MEFs or serum coating and in published naive hPSCs and fibroblast lines. Source data are available online for this figure.

 © The Author(s)

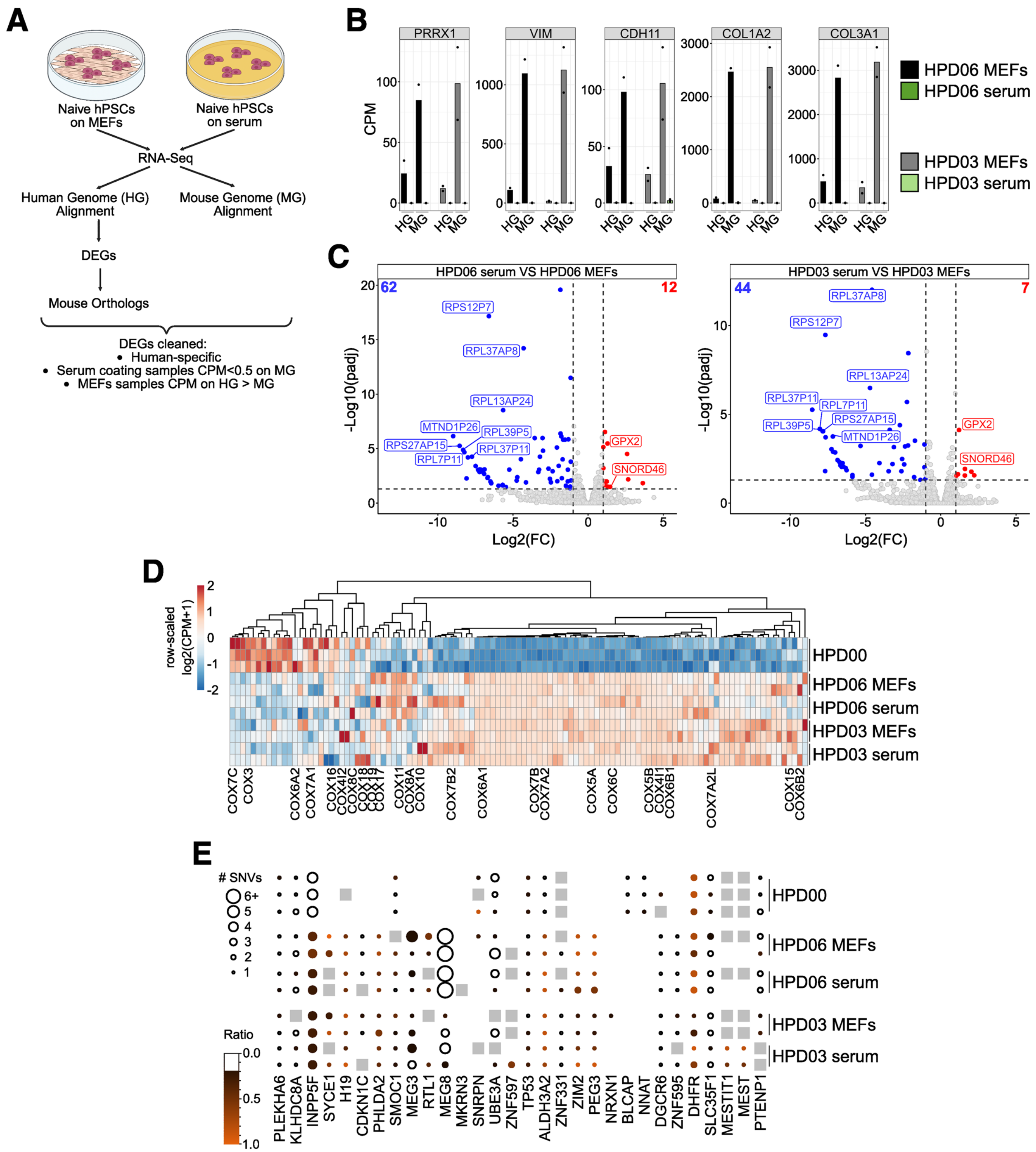
A
Naive hPSCs on MEFs
Naive hPSCs on serum
RNA-Seq
Human Genome (HG) Alignment
Mouse Genome (MG) Alignment
DEGs
Mouse Orthologs
DEGs cleaned:
• Human-specific
• Serum coating samples CPM<0.5 on MG
• MEFs samples CPM on HG > MG
B
PRRX1
VIM
CDH11
COL1A2
COL3A1
CPM
HPD06 MEFs
HPD06 serum
HPD03 MEFs
HPD03 serum
C
HPD06 serum VS HPD06 MEFs
HPD03 serum VS HPD03 MEFs
-Log10(padj)
Log2(FC)
62
12
44
7
RPS12P7
RPL37AP8
RPL13AP24
MTND1P26
RPL39P5
RPS27AP15
RPL37P11
RPL7P11
GPX2
SNORD46
D
row-scaled log2(CPM+1)
HPD00
HPD06 MEFs
HPD06 serum
HPD03 MEFs
HPD03 serum
COX7C
COX3
COX6A2
COX7A1
COX16
COX4I2
COX8C
COX18
COX19
COX11
COX8A
COX10
COX7B2
COX6A1
COX7B
COX7A2
COX5A
COX6C
COX5B
COX4I1
COX6B1
COX7A2L
COX15
COX6B2
E
SNVs
6+
5
4
3
2
1
Ratio
0.0
0.5
1.0
HPD00
HPD06 MEFs
HPD06 serum
HPD03 MEFs
HPD03 serum
PLEKHA6
KLHDC8A
INPP5F
SYCE1
H19
CDKN1C
PHLDA2
SMOC1
MEG3
RTL1
MEG8
MKRN3
SNRPN
UBE3A
ZNF597
TP53
ALDH3A2
ZNF331
ZIM2
PEG3
NRXN1
BLCAP
NNAT
DGCR6
ZNF595
DHFR
SLC35F1
MESTIT1
MEST
PTENP1

© The Author(s)

Figure EV3. Naive hPSCs on serum coating do not show expression of MEFs genes and retain the expression of OXPHOS and imprinted genes.

(**A**) Schematic representation of the pipeline followed for the evaluation and cleaning of MEFs DEGs between naive hPSCs stably cultured on serum coating and on MEFs. (**B**) Absolute expression (CPM) of some fibroblasts markers from Fig. 3D and other DEGs with similar behaviour identified between naive HPD06 and HPD03 hiPSCs stably cultured on serum coating and on MEFs. For each gene, expression is reported for each cell line and condition aligned against the human or mouse genome. (**C**) Volcano plot representing DEGs (|log2FC| > 1 and an adjusted *P*-value < 0.05, Benjamini–Hochberg adjustment, as indicated by dashed lines) between naive HPD06 (top) and HPD03 (bottom) hiPSCs stably cultured on MEFs and serum coating. Blue and red represent down- and up-regulated DEGs, respectively. Labels highlight the top 8 most significant shared down-regulated DEGs and the only two shared up-regulated DEGs between cell lines. (**D**) Heatmap of oxidative phosphorylation genes from the KEGG PATHWAY Database (https://www.genome.jp/kegg/pathway.html) and COX genes (Takashima et al, 2014) in primed HPD00 hiPSCs and naive HPD06 and HPD03 hiPSCs stably cultured on MEFs or serum coating. (**E**) BrewerIX gene summary panel results on RNA-Seq data from primed HPD00 hiPSCs and naive HPD06 and HPD03 hiPSCs stably cultured on MEFs or serum coating. Empty dots indicate detected genes with no evidence of biallelic expression, grey squares indicate genes detected but not reaching the thresholds, and the absence of any symbol indicates that the gene was not detected. Source data are available online for this figure.

 © The Author(s)

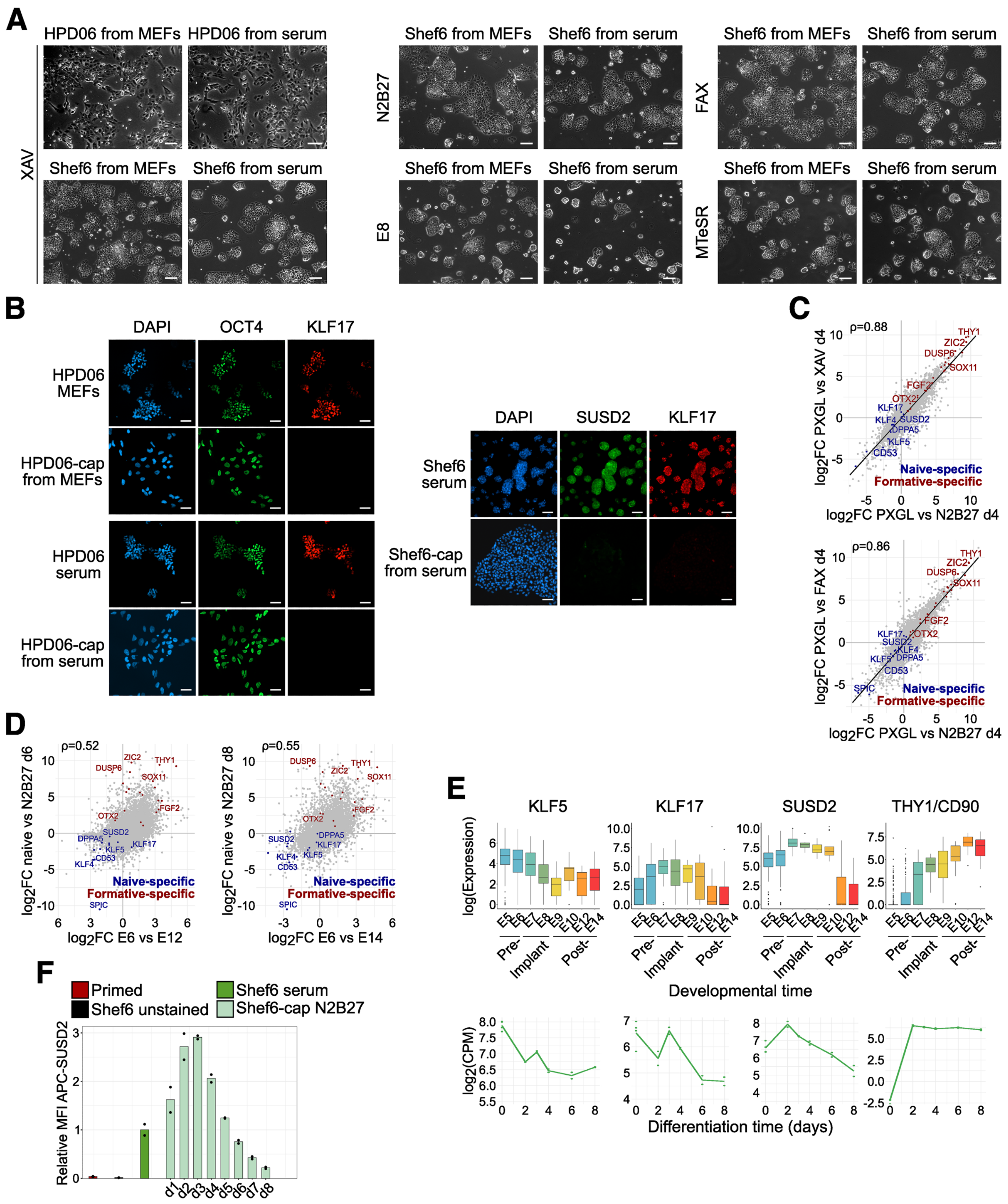
A
HPD06 from MEFs
HPD06 from serum
Shef6 from MEFs
Shef6 from serum
XAV
N2B27
FAX
E8
MTeSR
B
DAPI
OCT4
KLF17
HPD06 MEFs
HPD06-cap from MEFs
HPD06 serum
HPD06-cap from serum
SUSD2
Shef6 serum
Shef6-cap from serum
C
ρ=0.88
log2FC PXGL vs XAV d4
log2FC PXGL vs N2B27 d4
ρ=0.86
log2FC PXGL vs FAX d4
Naive-specific
Formative-specific
THY1
ZIC2
DUSP6
SOX11
FGF2
OTX2
KLF17
KLF4
SUSD2
DPPA5
KLF5
CD53
SPIC
D
ρ=0.52
log2FC naive vs N2B27 d6
log2FC E6 vs E12
ρ=0.55
log2FC naive vs N2B27 d8
log2FC E6 vs E14
E
KLF5
KLF17
SUSD2
THY1/CD90
log(Expression)
Pre-
Implant
Post-
Developmental time
log2(CPM)
Differentiation time (days)
F
Primed
Shef6 unstained
Shef6 serum
Shef6-cap N2B27
Relative MFI APC-SUSD2
d1
d2
d3
d4
d5
d6
d7
d8

Figure EV4. Feeder-free naive hPSCs can efficiently exit naïve pluripotency.

(**A**) Morphologies of HPD06 hiPSCs and Shef6 hESCs capacitated for 10 days from naive lines stably cultured on MEFs or serum coating under different media compositions. Scale bars: 100 µm. Representative images of two independent experiments are shown. (**B**) Immunostaining for general (OCT4) and naive (KLF17 and SUSD2) pluripotency markers of HPD06 hiPSCs and Shef6 hESCs capacitated for 10 days from naive lines stably cultured on MEFs or serum coating. Scale bars: 100 µm. Representative images of two independent experiments are shown. (**C**) Scatter Plots showing log2 fold changes (log2FC) of naive Shef6 hESCs stably cultured on serum coating differentiated for 4 days in unsupplemented N2B27, N2B27 supplemented with XAV, or N2B27 supplemented with XAV, FGF2, and Activin A (FAX) compared to the naive state. Technical replicates include $n = 6$ from two independent experiments for d0, $n = 4$ from two independent experiments for N2B27 at day 4, and $n = 2$ for all other combinations of conditions and time points. Data was filtered for the 9376 DEGs ($|\text{log2FC}| > 1$, padj < 0.05) identified in any comparison with the naive state. Selected naive- and primed-specific markers are labelled. Spearman correlation (ρ, $n = 9376$) is overlaid, with a linear regression line for visualisation fitted. (**D**) Scatter Plots showing log2 fold changes (log2FC) of naive Shef6 hESCs stably cultured on serum coating in the naive state versus differentiation for 6 or 8 days in N2B27, compared to corresponding developmental times (E6 vs. E12 or E14) from the human embryonic reference dataset (subset for embryonic lineages only) (Petropoulos et al, 2016). Technical replicates include $n = 4$ from two independent experiments for d0 and $n = 2$ for all other combinations of conditions and time points. Data was filtered for the 8508 DEGs ($|\text{log2FC}| > 1$, padj < 0.05) identified in any comparison between naive hESCs in PXGL and any differentiation timepoint in N2B27, as well as between E5 or E6 and any later embryonic day until E14. Spearman correlation (ρ, $n = 8508$) is overlaid, with selected naive- and primed-specific genes labelled. (**E**) Gene expression of naive (*KLF5*, *KLF17* and *SUSD2*), and primed (*THY1*) pluripotency markers in the human embryonic reference dataset (subset for embryonic lineages only, created with the https://petropoulos-lanner-labs.clintec.ki.se/shinys/app/ShinyEmbryoRef app applying default parameters and subsetting based on Reannotation (Prelineage, ICM, Epiblast only from (Petropoulos et al, 2016)) and for naive Shef6 hESCs stably cultured on serum coating differentiated for 6–8 days in N2B27, measured by RNA-seq. Lines indicate inferred trends based on mean values, with $n = 4$ replicates from 2 independent experiments for d0 and $n = 2$ technical replicates for any other time point shown as dots. (**F**) Expression of the naive-specific surface marker SUSD2 in naive Shef6 hESCs stably cultured on serum coating during capacitation for 8 days in N2B27, measured by flow cytometry using an APC-conjugated anti-SUSD2 antibody. The y-axis represents the relative median fluorescence intensity (MFI) normalised to the naive state. Bars represent the mean ± SEM of $n = 3$ technical replicates shown as dots for primed hPSCs. Technical replicates from $n = 1$ independent experiment for naive hPSCs and capacitated cells at different timepoints are shown as dots. Source data are available online for this figure.

 © The Author(s)

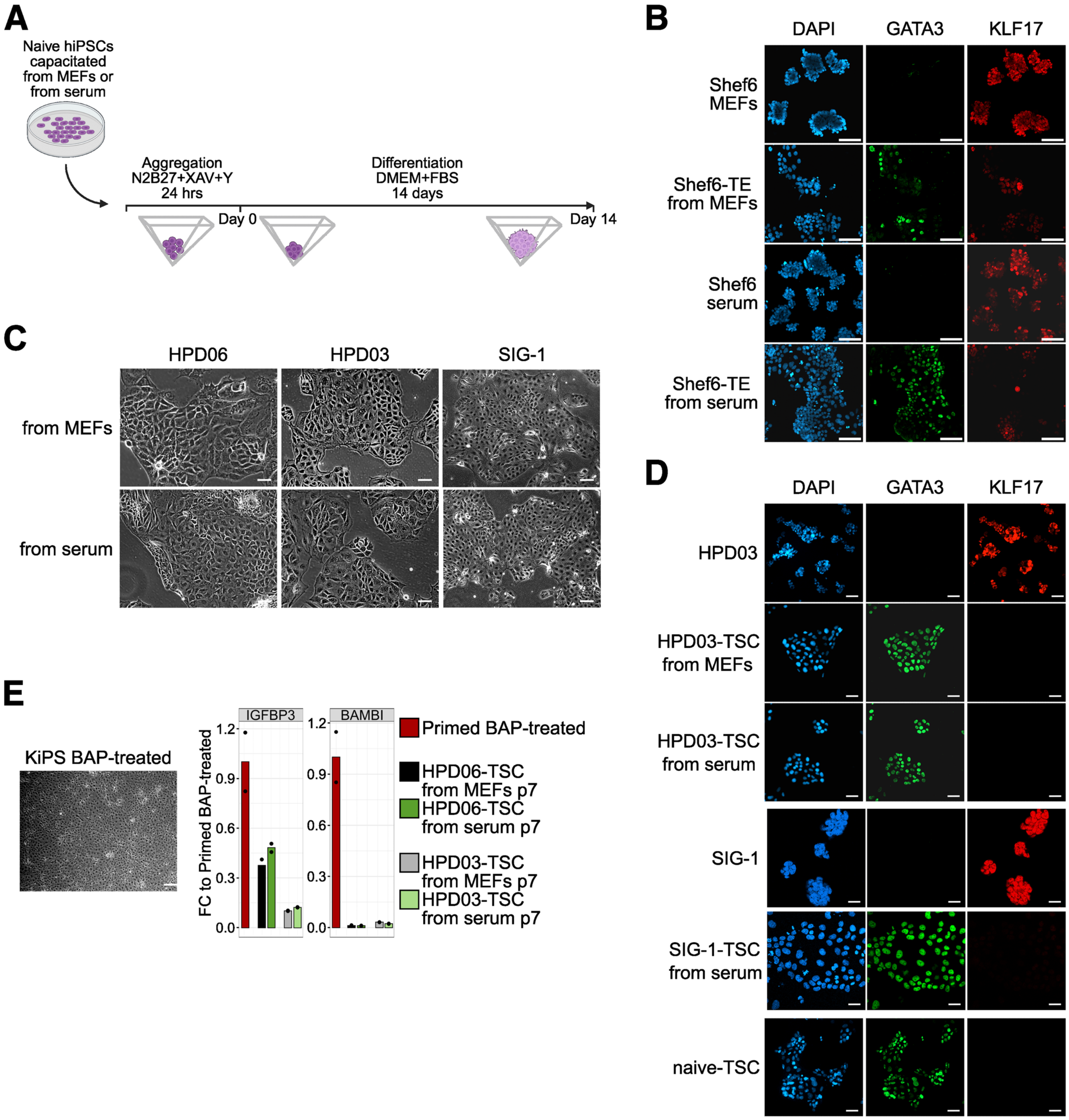

Figure EV5. Feeder-free naive hPSCs generate bona fide extraembryonic lineages.

(**A**) Schematic representation of the experimental setting for the EBs differentiation of naive HPD06 hiPSCs capacitated from MEFs or serum coating. (**B**) Immunostaining for TE/TSCs (GATA3) and naive pluripotency (KLF17) markers after 5 days of TE differentiation from naive Shef6 hESCs stably cultured on MEFs or serum coating. Scale bars: 100 µm. Representative images of two independent experiments are shown. (**C**) Morphologies of TSCs derived from naive HPD06, HPD03 and SIG-1 hiPSCs cultured on MEFs or serum coating. Scale bars: 100 µm. Representative images of two independent experiments are shown. (**D**) Immunostaining for TE/TSCs (GATA3) and naive pluripotency (KLF17) markers in TSCs derived from naive HPD03 and SIG-1 hiPSCs stably cultured on MEFs or serum coating. Scale bars: 100 µm. Representative images of two independent experiments are shown. (**E**) Left: Morphology of KiPS primed hiPSCs treated with BAP medium for 4 days. Scale bar: 100 µm. Right: Gene expression analysis by RT-qPCR of amnion markers (*IGFBP3* and *BAMBI*) in TSCs derived from naive HPD06 and HPD03 hiPSCs stably cultured on MEFs or serum coating. Technical replicates from $n = 2$ independent experiments are shown as dots. Source data are available online for this figure.

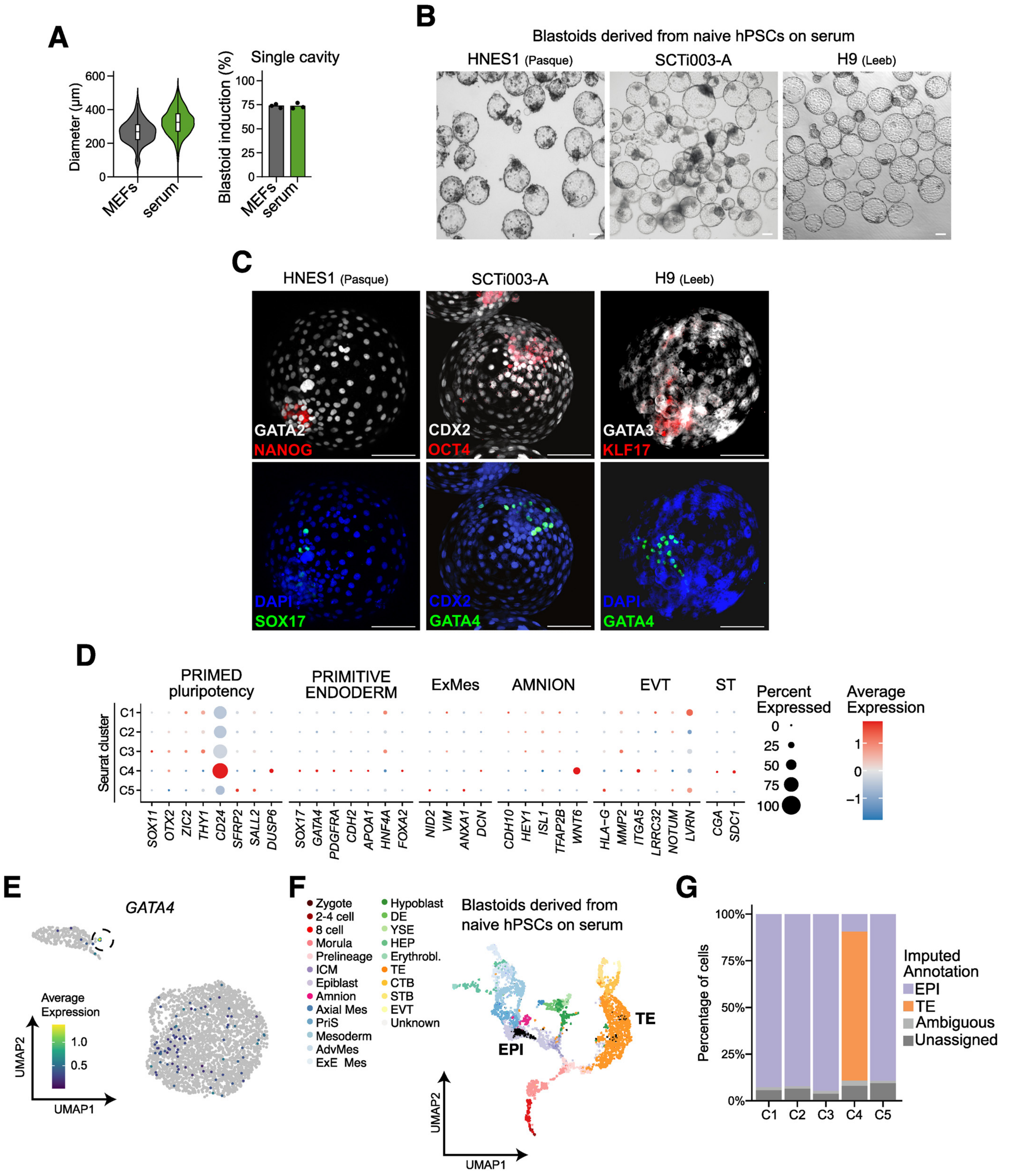
A
Single cavity
Diameter (µm)
Blastoid induction (%)
MEFs
serum
B
Blastoids derived from naive hPSCs on serum
HNES1 (Pasque)
SCTi003-A
H9 (Leeb)
C
HNES1 (Pasque)
SCTi003-A
H9 (Leeb)
GATA2
NANOG
CDX2
OCT4
GATA3
KLF17
DAPI
SOX17
CDX2
GATA4
DAPI
GATA4
D
PRIMED pluripotency
PRIMITIVE ENDODERM
ExMes
AMNION
EVT
ST
Percent Expressed
Average Expression
Seurat cluster
C1
C2
C3
C4
C5
SOX11
OTX2
ZIC2
THY1
CD24
SFRP2
SALL2
DUSP6
SOX17
GATA4
PDGFRA
CDH2
APOA1
HNF4A
FOXA2
NID2
VIM
ANXA1
DCN
CDH10
HEY1
ISL1
TFAP2B
WNT6
HLA-G
MMP2
ITGA5
LRRC32
NOTUM
LVRN
CGA
SDC1
E
GATA4
Average Expression
UMAP2
UMAP1
F
Zygote
2-4 cell
8 cell
Morula
Prelineage
ICM
Epiblast
Amnion
Axial Mes
PriS
Mesoderm
AdvMes
ExE Mes
Hypoblast
DE
YSE
HEP
Erythrobl.
TE
CTB
STB
EVT
Unknown
Blastoids derived from naive hPSCs on serum
EPI
TE
UMAP2
UMAP1
G
Percentage of cells
Imputed Annotation
EPI
TE
Ambiguous
Unassigned
C1
C2
C3
C4
C5

 © The Author(s)

Figure EV6. Blastoids from feeder-free naive hPSCs are transcriptionally similar to pre-implantation human embryos.

(**A**) Quantification of blastoid diameter (left) and blastoids with a single cavity (right) in structures formed from naive H9 hESCs cultured on MEFs or serum coating. Left: Data is presented as violin plots with the median, 25, 75 percentiles (± min-max) of $n = 407$ (MEFs) and $n = 806$ (serum coating) across $n = 3$ independent experiments. Right: Data shows the mean percentages of technical replicates from $n = 3$ independent experiments for naive H9 hESCs. (**B**) Brightfield images of blastoids induced from naive HNES1 and H9 hESCs, and SCTi003-A hiPSCs cultured on serum coating. Scale bar: 100 µm. (**C**) Immunostaining of blastoids derived from naive HNES1 and H9 hESCs and naive SCTi003-A hiPSCs cultured on serum coating. Blastoids were stained for TE (GATA2, CDX2 or GATA3), PrE (SOX17 or GATA4) and EPI (NANOG, OCT4 or KLF17) markers. Shown is the maximum projection. Scale bars: 100 µm. (**D**) Expression of selected lineage-specific marker genes across Seurat clusters from Fig. 6C. The size of the dots represents the proportion of cells in the indicated group expressing the given gene, and colour encodes the scaled average expression. (**E**) UMAP plot from scRNA-seq analysis as in Fig. 6C. Cells are coloured according to *GATA4* expression ($n = 3260$). (**F**) UMAP projection of in vitro d5 blastoids from naive H9 hESCs cultured on serum coating for 4 passages on the human pre-implantation and post-implantation embryos with annotation for scRNA-seq data integration from (Zhao et al, 2025). Cells are coloured by cell type, and black dots show neighbourhoods of in vitro-generated cells projected onto a reference UMAP. (**G**) Imputed cell annotation across different Seurat clusters as in Fig. 6F. Unassigned and ambiguous labels refer to cells with either none or with more than two imputed stages, respectively. Source data are available online for this figure.

© The Author(s)
